# ACC-BLA functional connectivity disruption in allergic inflammation is associated with anxiety

**DOI:** 10.1038/s41598-022-06748-w

**Published:** 2022-02-17

**Authors:** Leila Gholami-Mahtaj, Morteza Mooziri, Kolsoum Dehdar, Maryam Abdolsamadi, Morteza Salimi, Mohammad Reza Raoufy

**Affiliations:** 1 Department of Physiology, Faculty of Medical Sciences, Tarbiat Modares University, Tehran, Iran; 2 School of Medicine, Zahedan University of Medical Sciences, Zahedan, Iran; 3 Department of Mathematics, Faculty of Science, Islamic Azad University-North Tehran Branch, Tehran, Iran; 4 Institute for Brain Sciences and Cognition, Faculty of Medical Sciences, Tarbiat Modares University, Tehran, Iran

**Keywords:** Neuroscience, Physiology

## Abstract

Allergic asthma is a chronic inflammatory respiratory disease. Psychiatric disorders, including anxiety are associated with poorer treatment response and disease control in asthmatic patients. To date, there is no experimental evidence describing the role of peripheral inflammation on the oscillatory activities in the anterior cingulate cortex (ACC) and basolateral amygdala (BLA), two major brain structures modulating anxiety. In the present work we evaluated lung and brain inflammatory responses, anxiety-like behavior, in association with oscillatory features of the ACC-BLA circuit in an animal model of allergic inflammation. Our data showed that allergic inflammation induced anxiety-like behavior and reactivation of microglia and astrocytes in ACC and BLA. Allergic inflammation also enhanced neuronal activities and functional connectivity of the ACC-BLA circuit which were correlated with the level of anxiety. Together, we suggest that disruption in the dynamic oscillatory activities of the ACC-BLA circuit, maybe due to regional inflammation, is an underlying mechanism of allergic asthma-induced anxiety-like behavior. Our findings could pave the way for better understanding the neuro-pathophysiology of the psychiatric disorders observed in asthmatic patients, possibly leading to develop novel treatment strategies.

## Introduction

Allergic asthma is a common chronic inflammatory respiratory disease in which reversible narrowing of the airways causes major symptoms like wheezing, shortness of breath, and coughing^1^. Among the serious complications attributed to asthma, studies have noted that psychological factors including depression and anxiety can affect patient outcomes^2,3^. A meta-analysis study estimated the pooled prevalence of anxiety disorders to be almost three times higher in youth with asthma, compared to the healthy youth^2^. It is also shown that asthmatic patients with anxiety have poorer treatment response and disease control leading to more emergency department visits^3,4^. Such observations highlight a link between asthma and functional brain alterations, yet the precise mechanisms by which allergic asthma causes emotional disturbances remain to be demonstrated.

Allergic inflammation is associated with disruptions in neural network activities, particularly brain regions involved in cognitive performances^5,6^. In this regard, there is a body of evidence elucidating the link between allergic inflammation and alterations in brain areas governing anxiety and emotional reactivity^6–8^. For instance, as we have previously shown, allergic asthma induces anxiety-like behavior in association with distraction in the medial prefrontal cortex (mPFC)-amygdala circuit^7^. Besides, histological studies indicated that these effects could be mediated by immune and molecular changes in different brain areas related to emotions^7,9^. As a specific example, anxiety-like behavior in allergic rhinitis is associated with increased production of T helper (Th) cytokines in the mPFC and olfactory bulbs^9^. Dysfunction of the circuit between these two regions, following allergic inflammation, induces anxiety-like behavior^6^. Hence, it is a quite common idea that allergen promotes inflammatory reactions in brain, and disrupt the neural activity of regions that actively form emotions, resulting in induction of anxiety-like behavior.

Several brain regions are noted to have a role in anxiety-related information processing^10^. In particular, preceding works introduce the basolateral amygdala (BLA) as one of the key brain regions for the expression of anxiety, which signals the presence of threat and salience information^10–12^. Expression of anxiety involves other brain regions including several cortical areas, forming the notion of cortico-amygdala circuitries^11^. Among such regions, the anterior cingulate cortex (ACC), a subregion of the mPFC with extensive connection to BLA, is shown to have a critical role in the regulation of anxiety^13–16^. Human imaging studies propose that anxiety is likely related to an ACC dysfunction^14,17^. In this line, altered activity in the ACC has been demonstrated in anxiety disorders like post-traumatic stress disorder and generalized anxiety disorder^18,19^. Thus, ACC may act as a higher structure to either directly or indirectly integrate anxiety signals from BLA.

There is experimental evidence indicating that anatomical and functional connections between ACC and BLA play an important role in anxiety expression and fear perception^13,14,20,21^. Besides, altered functional connectivity in the ACC-amygdala circuit is characterized to be associated with anxiety^11^. The top-down and bottom-up interactions in the cortico-amygdala circuitries represent high and low anxiety-related behaviors^7,11^. Accordingly, distorted bottom-up connections and/or top-down regulation in the ACC-amygdala circuit may lead to states of high anxiety^11,22^. Therefore, altered ACC-BLA signaling could be an underlying mechanism associated with anxiety disorders.

Taken together, the ACC and BLA form a circuit that seems to have a pivotal role in the expression of emotions and abnormal oscillatory activities of this circuit might contribute to the behavioral expression of anxiety. Furthermore, ACC and amygdala are important to brain structures whose activations are amplified in dyspnea, which is a common condition seen in asthma^23,24^. However, the extent to which allergic asthma causes network dysfunctions in the ACC-BLA circuit concerning anxiety remains an open question. In the present investigation, we hypothesized that allergen exposure induces inflammatory responses in ACC and BLA which in turn disrupts regional and inter-regional oscillatory activities of this circuit in association with enhancement of anxiety-like behavior. Therefore, we used a combination of behavioral experiments and electrophysiological recordings to assess anxiety-like behavior and resting-state oscillatory activities within the ACC-BLA circuit in an animal model of allergic asthma.

## Results

### Allergen induces inflammatory responses in the lung and brain

**Figure 1:**
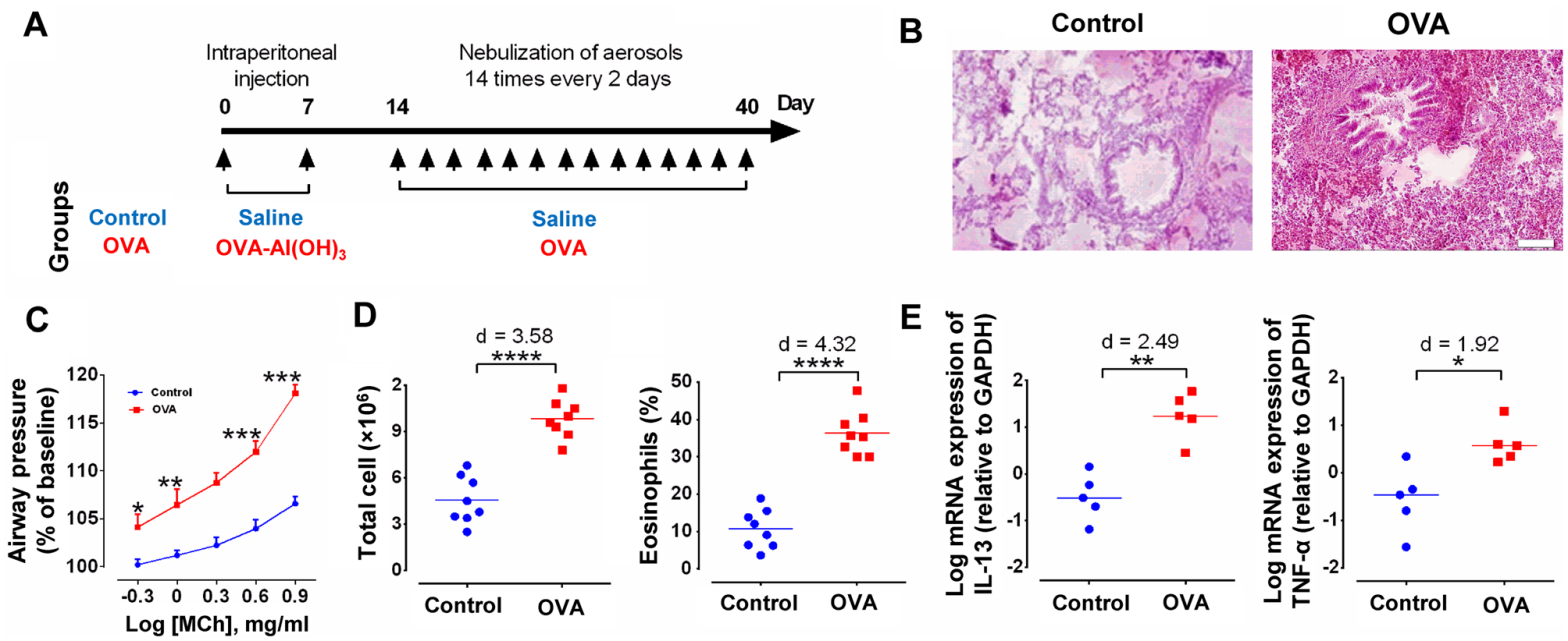
Study design and inflammatory responses in the lung. (**A**) Timeline of the study design. The rats received 2 intraperitoneal injections of saline or OVA-Al(OH)_3_ on day 0 and day 7, and 14 inhalation exposures (for 30 min, every 2 days, from day 14 to day 40) with aerosols of saline or OVA solution (2% wt/vol). (**B**) Lung H&E staining to recognize peribronchovascular inflammatory cell infiltration. Scale bar represents 50 μm. (**C**) Airway hyperresponsiveness following increasing doses of methacholine. Data are presented as percent of baseline, expressed as mean ± SEM, and analyzed by two-way repeated measures ANOVA, with the Bonferroni post-hoc test, n = 8 per group. (**D**) Total inflammatory cells (left panel) and eosinophils (right panel) in BALF. The horizontal bars represent mean values. Data were analyzed by t-test, n = 8 per group. (**E**) mRNA expression of IL-13 (left panel) and TNF-α (right panel) in the lung. The horizontal bars represent median values. Data were analyzed by Mann–Whitney test, n = 5 per group. *p < 0.05, **p < 0.01,***p < 0.001 and ****p < 0.0001 compared to control. OVA, ovalbumin; ACC, anterior cingulate cortex; BLA, basolateral amygdala.

To induce allergic lung inflammation, sensitized rats were exposed to Ovalbumin (OVA) according to our previous study (Fig. 1A)^7^. Histopathological results showed that allergic inflammation induced an intense peribronchovascular inflammatory infiltrate in the lungs of sensitized rats (Fig. 1B). OVA challenge increased airway responsiveness to cumulative doses of methacholine (MCh) in sensitized rats (p < 0.001) (Fig. 1C). In the bronchoalveolar lavage fluid (BALF), the level of total inflammatory cells and eosinophils were enhanced in OVA rats (p < 0.001, d = 4.58; p < 0.001, d = 6.32, respectively) (Fig. 1D). Also, exposure to OVA significantly augmented the mRNA expression of IL-13 (P < 0.01, d = 2.49) and TNF-α (P < 0.05, d = 1.92) in the lung tissue (Fig. 1E).

**Figure 2:**
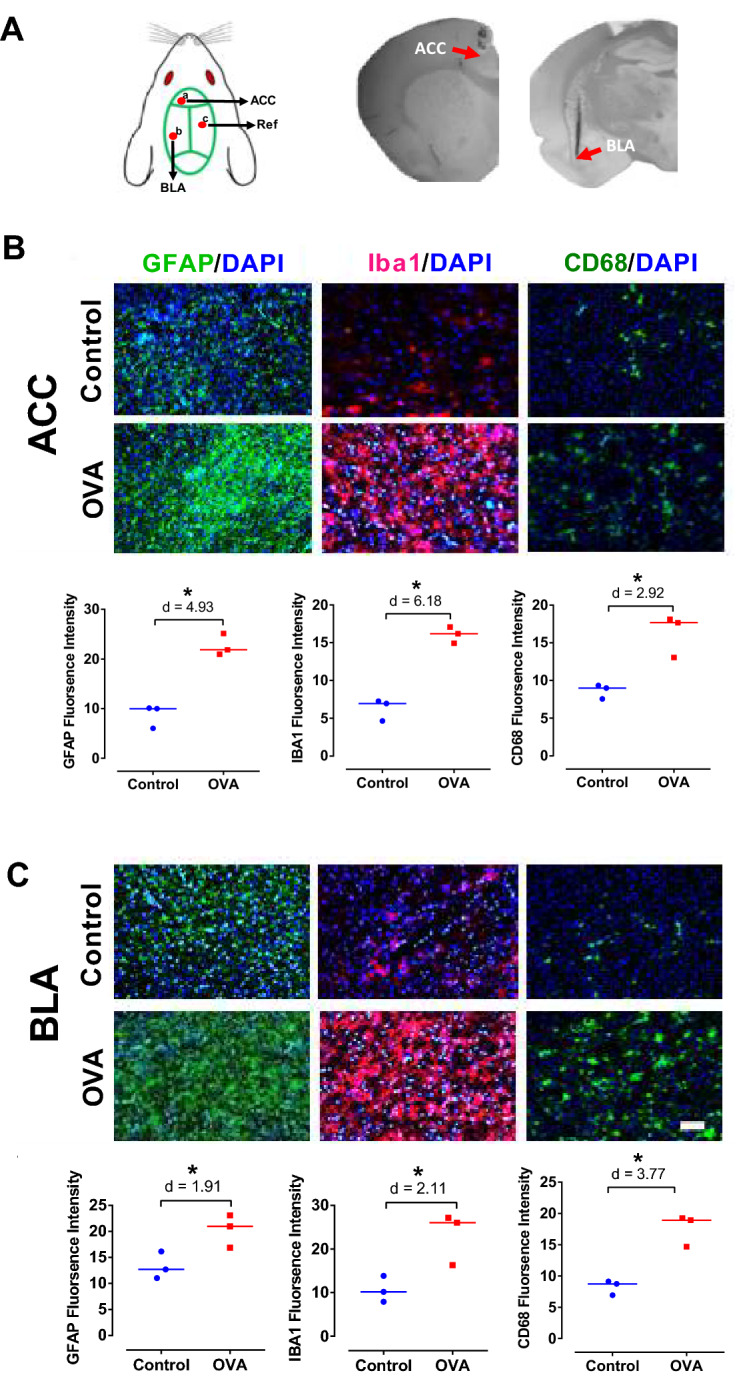
Inflammatory responses in brain. (**A**) Schematic illustration of electrode implantation sites (left panel) and histological verification of recording sites in ACC and BLA (right panel). (**B, C**) Immunofluorescence reactivity of microglia (CD68 and Iba1) and astrocytes (GFAP) counterstained with DAPI in ACC (**B**) and BLA (**C**), represented in raw images (upper panels) as well as quantitative values (lower panels). Scale bar represents 50 μm. The horizontal bars represent median values. Data were analyzed by Mann–Whitney test, n = 3 per group. *p < 0.05 compared to control. OVA, ovalbumin; ACC, anterior cingulate cortex; BLA, basolateral amygdala.

Figure 2 shows histological verification of electrode implantation sites (Fig. 2A) and immunofluorescence staining of ACC and BLA. Immunofluorescence staining of brain sections displayed that allergen enhanced microglia in ACC (Iba1 + microglia: p < 0.05, d = 6.18; CD68 expression: p < 0.05, d = 2.92) and BLA (Iba1 + microglia: p < 0.05, d = 2.11; CD68 expression: p < 0.05, d = 3.77) (Fig. 2B, C). Also, astrocyte (GFAP) reactivation was enhanced in ACC (p < 0.05, d = 4.93) and BLA (p < 0.05, d = 1.91) of OVA animals compared to controls (Fig. 2B, C). Our data exhibited that allergen activates inflammatory mediators of the Th2 pathway, which in turn induces inflammation in the lung and brain.

### Allergic inflammation induces anxiety-like behavior

**Figure 3:**
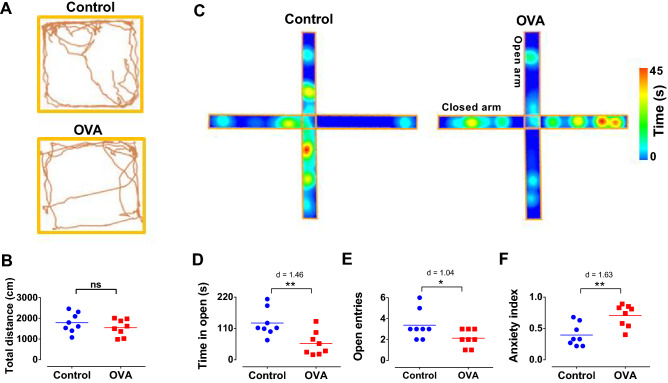
Locomotor activity and anxiety-like behavior. (**A**) Representative traces of total distance traveled for control and OVA rats in the open field test. (**B**) Quantification of total distance traveled for in the open field test. (**C**) Sample heat map of elevated plus-maze exploration for the control and OVA groups. Warmer colors illustrate increased time spent on the segment. (**D-F**) Quantification of the time spent on open arms (**D**), the number of open entries (**E**), and anxiety index (**F**). The horizontal bars represent mean values. Data were analyzed by t-test, n = 8 per group. *p < 0.05, **p < 0.01 compared to control. OVA, ovalbumin.

Analysis of total distance traveled in the open-field test did not reveal a significant difference in locomotor activity between groups (Fig. 3A, B). It rules out a possible influence of locomotor disability due to allergic inflammation. In the elevated plus-maze test, allergen exposure in sensitized rats significantly reduced the time spent in the open arms (p < 0.01, d = 1.46) and the number of open arms entries (p < 0.05, d = 1.04) (Fig. 3C-E), leading to increased anxiety index (p < 0.01, d = 1.63) (Fig. 3F).

### Allergic inflammation-induced anxiety-like behavior is correlated with enhancement of ACC and BLA activity

**Figure 4:**
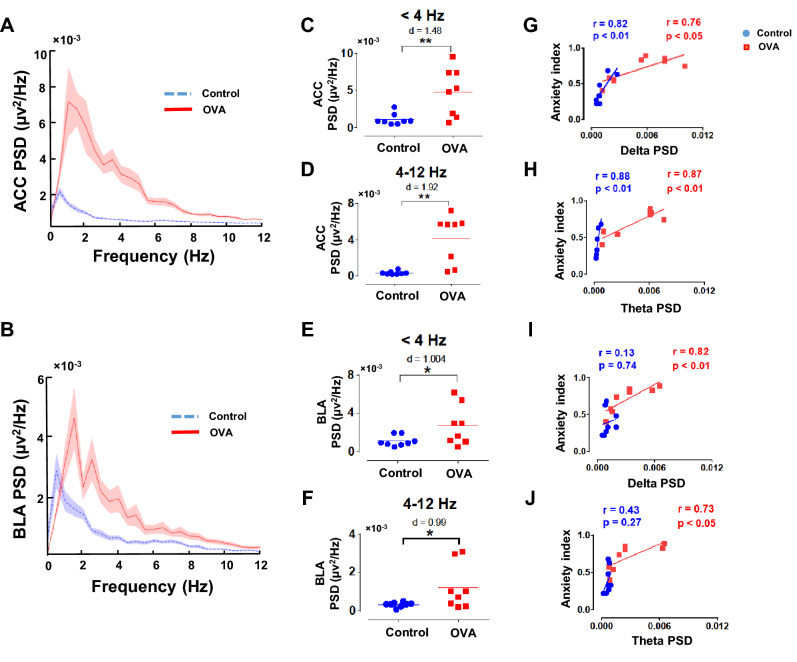
ACC and BLA neural activities. (**A, B**) PSD of recordings in the ACC (**A**) and BLA (**B**). Shaded areas show SEM. (**C-F**) Averaged PSD of ACC (**C, D**) and BLA (**E, F**) at delta (upper panels) and theta (lower panels) frequency bands. The horizontal bars indicate mean values. (**G-J**) Correlation between levels of anxiety and PSD of ACC (**G, H**) and BLA (**I, J**) at delta (upper panels) and theta (lower panels) frequency bands. Correlation coefficient (r) was performed using Pearson correlation coefficient analysis. Data were analyzed by t-test, n = 8 per group. *p < 0.05, **p < 0.01 compared to control. OVA, ovalbumin; ACC, anterior cingulate cortex; BLA, basolateral amygdala; PSD, power spectral density.

We used local field potential (LFP) recordings in awake rats to assess oscillatory activities of the ACC and BLA at delta (< 4 Hz) and theta (4–12 Hz) frequencies in Control and OVA groups (Fig. 4A, B). Allergen exposure in sensitized rats significantly enhanced delta and theta activity in ACC (p < 0.01, d = 1.48; p < 0.01, d = 1.92, respectively) and BLA (p < 0.05, d = 1.004; p < 0.05, d = 0.99, respectively) (Fig. 4C-F). In addition, the anxiety index was positively correlated with ACC power spectral density (PSD) at delta (Control: r = 0.82, p < 0.01; OVA: r = 0.78, p < 0.05) and theta (Control: r = 0.88, p < 0.01; OVA: r = 0.87, p < 0.01) frequencies (Fig. 4G, H). This correlation was also observed in BLA only for the OVA group (r = 0.82, p < 0.01; r = 0.73, p < 0.05, respectively) (FIg. 4I, J). Therefore, allergen increases ACC and BLA activities which are positively correlated with anxiety-like behavior.

### Allergic inflammation-induced anxiety-like behavior is correlated with enhancement of ACC-BLA synchrony

**Figure 5:**
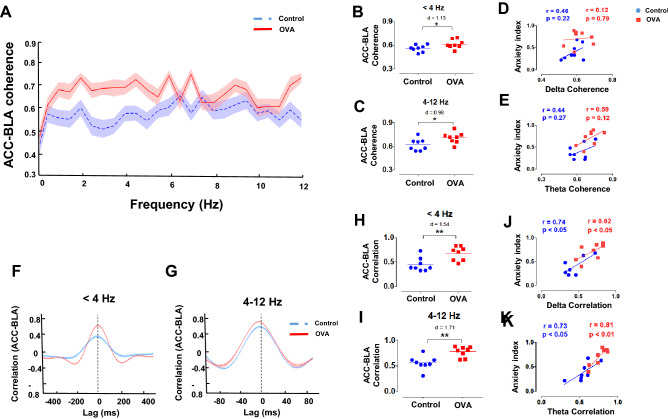
ACC-BLA functional connectivity. (**A, F, G**) Coherence spectra (**A**) and cross-correlation (**F, G**) between ACC and BLA. Shaded regions demonstrate SEM. (**B, C, H, I**) Values of coherence (**B, C**) and correlation (**H, I**) between ACC and BLA at delta (upper panels) and theta (lower panels) frequency bands. The horizontal bars indicate mean values. (**D, E, J, K**) Correlation between levels of anxiety and ACC-BLA coherence (**D, E**) and cross-correlation (**J, K**) at delta (upper panels) and theta (lower panels) frequency bands. Correlation coefficient (r) was performed using Pearson correlation coefficient analysis. Data were analyzed by t-test, n = 8 per group. *p < 0.05, **p < 0.01 compared to control. OVA, ovalbumin; ACC, anterior cingulate cortex; BLA, the basolateral amygdala.

Since there is experimental evidence indicating that functional connectivity between ACC and BLA play an important role in anxiety expression^21^, we studied whether allergic inflammation changes ACC-BLA coupling. Coherence analysis of the simultaneous recordings of ACC and BLA signals showed significantly greater values for OVA rats at delta and theta bands (p < 0.05, d = 1.13; p < 0.05, d = 0.98, respectively) (Fig. 5A-C). However, there was no significant correlation between ACC-BLA coherence and anxiety index (Fig. 5D, E).

We also used cross-correlation analysis to further assess the synchrony of the ACC-BLA circuit. In OVA animals, ACC-BLA correlation was enhanced for delta and theta frequencies (p < 0.01, d = 1.54; p < 0.05, d = 1.71, respectively) (Fig. 5F-I). Importantly, we observed a positive correlation between anxiety index and ACC-BLA synchrony at delta (Control: r = 0.74, p < 0.05; OVA: r = 0.62, p < 0.05) and theta frequencies (Control: r = 0.73, p < 0.05; OVA: r = 0.81, p < 0.01) (Fig. 5J, K). Our analyses demonstrated that allergen increases ACC-BLA coupling in association with enhancement of anxiety-like behavior.

### Allergic inflammation-induced anxiety-like behavior is correlated with enhancement of ACC-BLA phase-amplitude coupling

**Figure 6:**
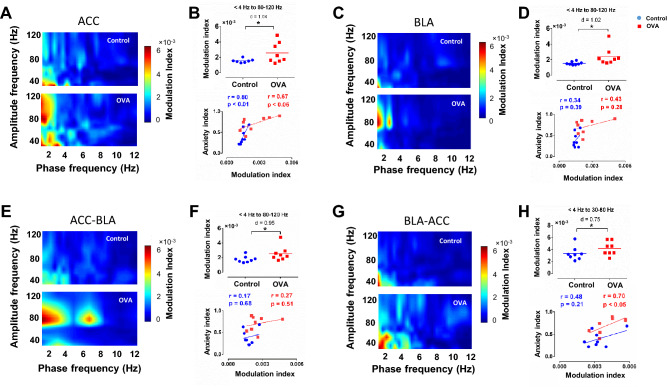
Phase-amplitude coupling within the ACC-BLA circuit. (**A, C, E, G**) Phase-amplitude comodugram of representative ACC (**A**), BLA (**C**), ACC-BLA (**E**), and BLA-ACC (**G**). (**B, D, F, H**; upper panels) Peak modulation index across phase frequencies computed for gamma amplitude in ACC (**B**), BLA (**D**), ACC-BLA (**F**), and BLA-ACC (**H**). The horizontal bars indicate mean values. (**B, D, F, H**; upper panels) Correlation between anxiety index and PAC of ACC (**B**), BLA (**D**), ACC-BLA (**F**), and BLA-ACC (**H**). Correlation coefficient (r) was performed using Pearson correlation coefficient analysis. Data were computed by t-test, n = 8 per group. *p < 0.05 compared to control. OVA, ovalbumin; ACC, anterior cingulate cortex; BLA, basolateral amygdala; PAC, phase-amplitude coupling.

Studies have clarified the importance of coupling between the phase of low-frequency oscillations and the amplitude of high-frequency oscillations during anxiety^25^. Here, we investigated whether delta/theta-gamma coupling of ACC and BLA was increased following allergic inflammation. Our analysis showed that allergen significantly enhanced the modulatory effect of delta phase on the regional gamma2 amplitude (80–120 Hz) in ACC (p < 0.05, d = 1.04) and BLA (p < 0.05, d = 1.02) (Fig. 6A-D). Pearson's correlation coefficient analysis displayed a positive correlation between anxiety index and PAC of ACC, but not BLA (Control: r = 0.80, p < 0.01; OVA: r = 0.67, p < 0.05) (Fig. 6B, D; lower panels). Our data showed that allergic inflammation-induced anxiety-like behavior is associated with increased regional PAC in ACC and BLA.

In addition, allergen exposure in sensitized rats significantly increased delta-gamma2 (p < 0.05, d = 0.95) and theta-gamma2 (p < 0.05, d = 0.78) phase-amplitude coupling (PAC) of ACC-BLA (Fig. 6E, F; theta-gamma2 results are not shown). A positive correlation was found between anxiety index and ACC-BLA theta-gamma2 coupling (Control: r = 0.73, p < 0.05; OVA: r = 0.81, p < 0.01) (data not shown). Allergen also enhanced delta-gamma1 (30–80 Hz) PAC of BLA-ACC (p < 0.05, d = 0.75) (Fig. 6G, H) which was positively correlated with anxiety index (r = 0.70, p < 0.05) (Fig. 6H; lower panel). These findings revealed that enhanced inter-regional PAC of the ACC-BLA circuit is associated with anxiety-like behavior induced by allergic inflammation.

## Discussion

The ACC and BLA are key brain regions in emotional processing, in particular anxiety^10,14^. Pathological conditions induce anxiety-like behavior along with disruptions in the ACC-BLA oscillatory activities^26^. However, to date, there is no study describing the role of network interactions within the ACC-BLA circuit in allergic asthma-induced anxiety-like behavior. In the present study, we used behavioral assessments and electrophysiological recordings in awake immobile rats to evaluate the effects of allergic inflammation on the level of anxiety-like behavior and the ACC-BLA circuit oscillatory activities. Our results show that allergen stimulates inflammatory reactions in brain and increases activity and functional connectivity in the ACC-BLA circuit, which is correlated with enhanced anxiety-like behavior. Together, our data for the first time indicate that abnormal changes in the dynamic ACC-BLA oscillatory activities induced by allergic asthma may play a key role in the expression of anxiety-like behavior.

Allergic inflammation activates both local and systemic Th2 cells, which produce several inflammatory cytokines, including IL-13, and have a major role in the pathophysiology of allergic asthma^27^. On the other side, expression of anti-inflammatory cytokines, such as IL-37, are decreased in allergic conditions and exert a disinhibitory effect on Th2 secretory activity, resulting in hypersecretion of inflammatory cytokines by these cells^28^. The communication between the brain and immune system happens through different pathways, divided into two main branches, the neural and the humoral pathways^29^. In the neural pathway, primary afferent nerves are activated by allergens and cytokines in peripheral organs. In the case of allergic reactions in the lung, signal of activated vagal sensory fibers project to the nucleus tractus solitarius, and from there to several brain regions^29,30^. In the humoral pathway, peripherally produced pathogen-associated molecular patterns and cytokines infiltrate into brain tissue through choroid plexus and regions lacking the blood–brain barrier (BBB), known as circumventricular organs, and induce production and release of pro-inflammatory cytokines^29^. The release of immune mediators in the brain can stimulate neuronal activation through interactions with neurons and glia^7,29,31^. Specifically, Th2 cells are primed in peripheral organs and enter the brain by crossing BBB^32^. Subsequently, regional microglia and astrocytes, as antigen-presenting cells, stimulate Th2 inflammatory secretion by introducing the target antigen to these cells^32^. This pathway will consequently result in increased secretion of inflammatory cytokines, such as IL-13, in the brain^32^. Additionally, anti-inflammatory cytokines, such as IL-37, might have neuroprotective effects through targeting astrocytes and microglia and suppressing immunity responses such as those related to Th2^33–35^. The imbalance between inflammatory and anti-inflammatory cytokines promotes neuroinflammation and might damage the neuroprotective mechanisms existing within the brain^32,33^, which in turn might disturb normal brain function and behavior. Accordingly, the interaction between Th2 and its associated inflammatory profile, as well as the degree of correlation between local and peripheral inflammation is of interest in this setting which demands further work for the immunological pathways to be elucidated. Herein, we show that allergen exposure in sensitized rats leads to aggregation of inflammatory cells in lung, and activates astrocytes and microglia in the ACC and BLA. Our previous study has also shown that allergic condition induces microglia- and astrocyte-mediated inflammation in mPFC and amygdala in association with induction of anxiety-like behavior^7^. The influx of inflammatory cytokines into the paraventricular nucleus of the hypothalamus is reported to increase astrocyte reactivity, and their release of γ-aminobutyric acid in association with enhancement of anxiety-like behavior^36^. Besides, chronic mild stress induces anxiety-like behavior through hippocampal neuroinflammation mediated by microglial activation^37^. Consequently, activation of microglia and astrocytes enhances neuronal activity at different frequency bands, including delta, theta, and low-gamma^7,31^. These effects were reported in several brain regions that actively encode anxiety, in particular mPFC, amygdala, and hippocampus^7,31^. Altogether, we propose that peripherally presented allergens activate local astrocytes and microglia in ACC and BLA. These activated cells facilitate neuronal activity of the mentioned regions, which in turn may produce anxiety-like behavior. Future studies are needed to explore the induction of systemic inflammation following an allergic respiratory disorder and the subsequent inflammatory responses in the brain.

The BLA has an established role in the regulation of anxiety and is connected to several brain areas that process anxiety-related information^10,14^. Previous rodent investigations have indicated an association between increased BLA activity and anxiety-like behaviors^38,39^. Human studies provide further support that BLA hyperactivity accompanies anxiety-related behaviors^12^. In line with these observations, here we show that allergen-induced anxiety-like behavior is correlated with increased resting-state power of delta and theta frequencies in BLA. Furthermore, previous works suggest that higher levels of anxiety in an anxiogenic situation tend to accompany increased PAC in local cortical regions^40,41^. Therefore, we tried to investigate whether allergen exposure in sensitized rats could alter BLA delta/theta-gamma PAC. Our results show that allergic inflammation increased BLA delta-gamma2 PAC, suggesting that the local delta phase strongly modulates the amplitude of gamma2 frequencies in BLA. BLA PAC is believed to be a principal neural code of emotions and changes in the modulatory effect of BLA theta on regional gamma mediate the expression of fear and safety^25^. Together, BLA malfunction following allergic inflammation might be a potential neurobiological mechanism predisposing individuals to anxiety disorders.

In addition to BLA, human and animal studies have reported the involvement of ACC in anxiety-related behaviors^14^. The observed anxiolytic effects following surgical ablation or chemical inactivation of ACC^42,43^, anxiety-like consequences of the ACC hyperactivity^15^, and increased ACC activity in patients suffering from anxiety disorders^17^ support the idea that enhanced ACC activity is involved in anxiety-related information processing. In this case, our results exhibit that the increased power of ACC in delta and theta frequencies following allergic inflammation is correlated with anxiety-like behavior, both in control and allergic asthma animals. Previous works have shown increased cross-frequency coupling in frontal brain regions in anxious animals and humans^6,40^. A human electroencephalographic (EEG) recording study reports that individuals with anxiety have higher levels of coupling between the phase of slow waves and the power of fast waves in frontal regions^40^. It is also reported that allergic rhinitis induces anxiety-like behavior and increases mPFC PAC^6^. Thus, we tried to investigate whether allergic inflammation could change ACC PAC and whether such changes are correlated with anxiety-like behavior. Based on our results, allergic inflammation strongly increases local modulation of gamma2 amplitude by delta phase in ACC. Besides, this modulatory effect is positively correlated with the expression of anxiety-like behavior. The phase of an oscillation contains considerable physiological information which reflects the excitability state of a neuronal population, and cross-frequency interactions embracing the signal phase can determine different functions of the high frequency oscillations^44^. In this context, the PAC in a single region carries important information about how the two oscillations interact^44^. A possible explanation for the observed phenomenon is that slow oscillations may organize brain functions, including emotional behaviors, through adjusting the timing of neuronal units, although the neurophysiological explanations of such findings remain to be explored^44^. Overall, we propose that ACC plays a key role in processing anxiety-related information, and that abnormal ACC function due to allergic inflammation is likely to produce anxiety-related behaviors.

Experimental evidence provide anatomical and functional projections between ACC and BLA^13^. Former studies have indicated functional connectivity between frontal brain regions and the amygdala in anxious humans and animals^7,45^. In this line, the ongoing ACC-BLA communication is proven to be an important mechanism underlying aversive processing in pathological anxiety^16^. Along with these observations, here we report that allergic inflammation increases coherence and cross-correlation between ACC and BLA, exhibiting enhanced functional connectivity in this circuit; the increased ACC-BLA synchrony induced by allergic inflammation is correlated with expression of anxiety-like behavior. Preceding work indicates reduced connectivity between the amygdala and ACC in anxious children, whereas anxious adults showed more positive connectivity compared to the healthy control^11^. These results might indicate greater bottom-up processing in childhood or altered top-down regulation of amygdala by ACC in young adulthood^11^. Therefore, failure to establish proper ACC-BLA functional connectivity might be a predisposing factor for anxiety and potentially other psychopathologies^11^. Pathological conditions, in particular peripheral inflammation, induce inflammatory responses in different brain regions, which in turn alter the oscillatory activities of the involved regions, through the mechanisms discussed earlier^7,29^. These changes disrupt the functional connectivity of the networks and enhance the level of anxiety^7^. In line with previous studies, we suggest that the potentiated coupling between frontal brain regions and amygdala nuclei, including BLA, following allergic inflammation might be a neural code that adaptively increases vigilance towards threatening stimuli and anxiety expression. These changes might underly the improper level of anxiety observed in asthmatic patients. However, the precise functional connectivity of these regions during different anxiogenic contexts demands further investigations.

There is a body of evidence suggesting different modulatory mechanisms in the ACC-BLA circuit during emotional processing^13,20^. In this line, previous investigations propose that the BLA might detect threats, but ACC could be a key node in the expression of anxiety behaviors, integrating threat information to dynamically adjust behavioral response through synchronized activity with other brain regions, including BLA^16^. Moreover, the inactivation of ACC projections to the BLA significantly reduces generalized fear to a novel non-threatening context^20^, but enhances innate fear of predator odors^13^. Accordingly, we tried to investigate whether the bidirectional modulatory effects between ACC and BLA were changed following the induction of allergic inflammation. In this case, we found that allergic inflammation increases modulation of BLA gamma2 amplitude by ACC delta and theta phases. Moreover, the value of this modulation at theta phase is correlated with anxiety-like behavior. On the other direction, our results exhibited that allergic inflammation enhances modulation of ACC gamma1 amplitude by BLA delta phase which is correlated with expression of anxiety-like behavior. It is previously suggested that dysregulation in the top-down or bottom-up connections between ACC and amygdala may be associated with anxiety. Together, our results suggest that disruption of ACC-BLA top-down or bottom-up connections might have a key role in the expression of anxiety in allergic conditions.

This study provides valuable new insights into the neuro-pathophysiology of allergic asthma-induced anxiety. Here we report that allergic inflammation induces ACC-BLA network dysfunctions that are correlated with anxiety-like behavior. Such changes in the connectivity between different brain regions might eventually cause psychiatric disorders. Therefore, treating asthmatic patients should include interventions to prevent inflammatory reactions in brain which in turn reduces behavioral disorders. In addition to previous evidence, our findings further emphasize the importance of attention to psychiatric aspects of asthmatic patients.

There were some limitations in the current study, which need to be addressed. First, although previous studies indicated that OVA exposure produces a systemic inflammation leading to subsequent neuroinflammation and cognitive impairments^46,47^, the interaction between peripheral and central inflammatory modulators should receive further attention in future studies to discover the mechanisms by which asthma induces a systemic inflammatory condition which in turn triggers neuroinflammation. Second, it is a reasonable approach to use a positive control group validated for anxiety-like behavior in rodents, which provides the opportunity for a plausible comparison of the behavioral and neurophysiological findings of the study, to further confirm the results. Third, Further work using real-time LFP and single-unit recordings, at the same time that the animal explores the elevated plus-maze, together with more advanced neural manipulations can provide a deeper understanding of the contribution of the ACC-BLA circuit in the expression of anxiety-like behavior in allergic condition.

## Conclusion

Here we provide a mechanistic insight into how allergic inflammation induces anxiety-like behavior. Allergen exposure in sensitized rats induces inflammatory responses in ACC and BLA, which are key brain regions governing anxiety-related behaviors. Additionally, allergic inflammation increases BLA and ACC oscillatory activities and enhances ACC-BLA circuit functional connectivity. We suggest that allergic inflammation disrupts the top-down regulations and bottom-up connections in the ACC-BLA circuit. Our study might provide a window for development of treatment approaches aiming at reducing psychiatric disorders induced by allergic inflammation.

## Methods

### Animals

The experimental animals in this study were sixteen pathogen-free male Wistar rats weighing 80–100 gr with ages between 4–5 weeks and were obtained from Pasteur Institute (Tehran, Iran). They were housed in standard laboratory conditions (21 ± 2 °C and 12:12 h light/dark cycle), with free access to food and water. The rats weighed between 250 and 300 g (10–11 weeks) at the time of the experiments. All protocols were performed in agreement with the guidelines of the NIH Guide for the Care and Use of Laboratory Animals (2011), recommendations in the ARRIVE guidelines^48^ and approved by the “Ethics Committee of Faculty of Medical Sciences, Tarbiat Modares University” (IR.TMU.REC.1396.695).

### Experimental groups and sensitization protocol

Animals were randomly distributed into the control and allergic asthma (OVA) groups (n = 8 in each group). The experimental protocol in this study is as previously reported^7^: 2 intraperitoneal injections of either saline or OVA-Al(OH)_3_ [1 mg OVA (Grade III; Sigma) and 100 mg Al(OH)_3_, in 1 ml saline] on days 0 and 7, followed by 14 aerosol inhalations (for 30 min every 2 days from day 14 to day 40) containing either saline or OVA solution (2% wt/vol) (Fig. 1A).

### Assessment of airway responsiveness and lung inflammation

We assessed airway hyperresponsiveness and BALF analysis 72 h after the last aerosol exposure, as previously described^7,49,50^. In brief, rats were anaesthetized (1.5 g/kg urethane, intraperitoneally), tracheally cannulated, and ventilated with a volume-controlled ventilator (Harvard Apparatus, Holliston, MA) which was set on a tidal volume of 1.0 ml/100 g and a rate of 70 breaths/min. To prevent efforts against mechanical ventilation, intraperitoneal injection of pancuronium bromide (0.2 mg/kg) was done. After 5 min of ventilation and stabilization, animals received inhalation of physiological saline for 1 min, to measure the basal airway pressure. Cumulative doses of MCh (0.5-1-2-4-8 mg/ml in saline) were inhaled for 60 s at 5-min intervals to evaluate the airway hyperresponsiveness, which was defined as the percentage of airway pressure triggered by MCh to basal pressure.

For BALF analysis, 3 ml of sterile physiological saline was gently instilled and withdrawn into the syringe (3 times) through the endotracheal tube. Each sample was centrifuged, and the deposited cells were washed three times in the RPMI Medium. Total cell and eosinophils counts were performed on BAL cytospin samples prepared by cytocentrifugation (cytospin 3, Shandon Instruments, Pittsburgh, PA) and stained with Giemsa^51^.

To recognize inflammatory cells that infiltrated the airways, lungs were fixated in formalin 10% and 5-µm sections were obtained for hematoxylin and eosin (H&E) staining.

### Measurement of mRNA expression of cytokines

Real-time quantitative PCR (R-qPCR) was used to assess the mRNA expression of IL-13, as a Th2 cytokine commonly attributed to allergic diseases, as well as TNF-α, a well-known pro-inflammatory mediator^52,53^. We used GAPDH RNA as the internal control. To this aim, 100 mg of lower lobe of the right lung was dissected frozen in liquid nitrogen. The removed section was kept in − 80 degrees Celsius. A mortar and pestle was used to crush the frozen lung sections in liquid nitrogen. TRIzol reagent was used to extract the total RNA. Single-stranded cDNA synthesis was achieved by cDNA reverse transcription kit (Aryatous Biotech, Tehran, Iran). The following oligonucleotide primers were used in this experiment: IL-13 primer (forward primer: 5′-GAGCAACATCACACAAGACCAG-3′, reverse primer: 5′-TGGAGATGTTGGTCAGGGATT-3′), TNF-α (forward primer: 5′-ACCACGCTCTTCTGTCTACTG-3′, reverse primer: 5′-CTTGGTGGTTTGCTACGAC-3′, and the GAPDH primer as the control (forward primer: 5′ACGGCAAGTTCAACGGCACAG-3′, reverse primer: 5′-GACATACTCAGCACCAGCATCACC-3′).

PCR reactions was conducted by adding 3 μL of cDNA, 1 μL of Forward and Reverse oligonucleotide primers (10 pmol/ml), and 10 ml of SYBR Green master-mix reagent (Ampliqon, Denmark) in a final volume of 20 ml, in the following protocol: 10 min incubation at 95 °C, followed by 45 cycles of denaturation step at 95 °C for 45 s, annealing step at 60 °C for 45 s, extension step at 72 °C for 45 s, followed by a final extension step for 10 min at 72 °C.

### Allergic inflammatory responses in brain

Inflammatory responses in and brain was evaluated using immunofluorescence staining of brain sections, 72 h after the last aerosol exposure. For that, transcardial perfusion with cold phosphate-buffered saline (PBS) followed by 4% paraformaldehyde solution was done prior to brain extraction. Subsequently, the extracted brains were post-fixated with paraformaldehyde overnight at 4 °C and cryoprotected for 48 h in 30% sucrose. Eight-μm thick coronal serial sections were collected from the level of the ACC and BLA and incubated overnight at 4 °C with primary antibodies for anti-Iba1 (1:100, SC-98468), anti-GFAP (1:300, Z0334), anti-CD68 (1:200, ab955). Slides were washed with PBS and incubated with secondary antibody (goat anti-rabbit AlexaFluor®594; 1:1000, A-11036, goat anti-rabbit AlexaFluor®488; 1:1000, A-11008, goat anti-mouse AlexaFluor®488; 1:1000, A-11001) at room temperature for 1 h, followed by washing with PBS. Finally, the sections were counterstained with DAPI and images were taken using fluorescence microscopy (Olympus BX51 TRF, USA)^7^. We used at least four segments of the ACC or BLA from each animal for quantification. Images were obtained from random, non-overlapping, consecutive microscopic fields in 200 × magnification using an Olympus BX-51 microscope and DP72 camera (6 images/section, 4 sections/animals, 3 animals/group). Image J was use to analyze fluorescence intensity.

### Behavioral assessments

The open field and elevated plus-maze were used 24 h after the last aerosol exposure. The rats’ movements were recorded by a video camera and ANY-maze video tracking software was applied for graphical analysis. The open-field test is an ordinary rodent behavioral test that can evaluate locomotor activity by measuring the total distance traveled. In this experiment, each animal was placed individually in the center of a standard open field box (50 cm high, 75 × 75 cm) and allowed to explore for 5 min. The elevated plus-maze consists of two open arms (50 × 10 cm) and two closed arms (50 × 10 cm) with 40 cm height. The apparatus was 50 cm higher from the floor. Animals were placed in the center of the maze, facing one of the open arms. Time spent in open and closed arms as well as the number of open and closed entries were recorded for 5 min. Consequently, the anxiety index was estimated as follows^54^:

$$\mathrm{Anxiety\,\, Index}=1 - \frac{\frac{\mathrm{Open\,\, arm\,\, time}}{\mathrm{Test\,\, duration}}+ \frac{\mathrm{Open \,\,arms \,\,entries}}{\mathrm{Total\,\, number \,\,of \,\,entries}}}{2}$$

### Electrodes Implantation and electrophysiological recording

Rats were anesthetized with intraperitoneal injections of ketamine (100 mg/kg) and xylazine (10 mg/kg). We checked the depth of anesthesia by examining tail and pinch reflexes. Once assured of anesthesia induction, the animals were fixed in a stereotaxic frame (Narishige, Japan) for electrode implantation. Before surgery, 0.5 ml of lidocaine chlorhydrate 2% was subcutaneously injected into the scalp to achieve local anesthesia. A heating pad was used to maintain the animals’ body temperature at 37 °C during the surgical procedures. Stainless-steel recording electrodes (127 μm in diameter, A.M. system Inc., USA) were inserted unilaterally into ACC (AP: + 3 mm; L: 0.6 mm; DV: − 1.5 mm) and BLA (AP: − 2.5 mm, L: 5 mm, DV: − 7.4 mm) according to the rat brain atlas in stereotaxic coordinates^55^. We placed a stainless-steel screw at the right parietal bone as the reference. Antibiotic (tetracycline) was used for scalp skin disinfection. Electrodes were fixed to the rats’ skull using dental cement. After the surgery, buprenorphine (0.1 mg/kg) as an analgesic, and sterile saline (1.0 ml) for hydration were intraperitoneally injected. Finally, rats were placed on a warm heating pad for recovery.

One week after the surgery (24 h after the last aerosol exposure), LFPs were recorded for 5 min in the animal’s home cage. For this aim, the socket, secured to the animal’s head, was connected to a miniature buffer headstage with high-input impedance (BIODAC-A, TRITA Health Tec. Co., Tehran, Iran), through cables to a main AC coupled amplifier (1000 amplification) and the recording system (BIODAC-ESR18622, TRITA Health Tec. Co., Tehran, Iran). Spontaneous LFPs of ACC and BLA were recorded simultaneously (low-pass filtered < 500 Hz, digitized at 1 kHz), and collected for offline processing with custom-written MATLAB routines (The Mathworks, Inc.).

For histological verification of the electrode placements, animals were deeply anesthetized using an intraperitoneal injection of urethane (1.2 g/Kg). After extraction, the brains were fixated in 4% paraformaldehyde for 48 h. Fifty-micrometer coronal brain sections were cut and mounted on glass slides. Electrodes’ locations were confirmed with light microscopy.

### Electrophysiological data analysis

LFP data were computed from 60 s periods of awake immobility offline in MATLAB (The Mathworks, Inc.) using built-in and custom-written routines. To detect awake immobility states, we used a video tracker to record the animals’ movements. LFP time series were divided into 6 segments with 10 s length. Analyses were achieved on these segments, and the average of obtained data was considered for each animal. PSD of LFP signals was assessed with welch function using Welch’s periodogram (90% overlapping, 6 s Hamming windows). Coherence spectra were calculated for ACC and BLA with magnitude-squared coherence (mscohere function; 90% overlapping, 6 s Hamming windows). We computed cross-correlation values using xcorr function for filtered signals of delta (< 4 Hz) and theta (4–12 Hz).

PAC was computed as previously described^56^. The coupling between the phase of the delta (< 4 Hz) and theta (4–12 Hz) bands, and the amplitude of the gamma band between 30 and 120 Hz were analyzed. The delta/theta phases were binned into eighteen 20° intervals and were averaged for each phase bin corresponding to the gamma amplitude. Then, phase-amplitude modulation index (MI) calculated the divergence of the empirical phase-amplitude profile from the uniform distribution. The comodulation map was obtained with estimates of the MI between multiple band-filtered frequency pairs and indicates the results in a 2D pseudocolor plot.

### Statistical analysis

We used GraphPad Prism (GraphPad Software, San Diego, CA) for statistical analysis. To determine data normality, we applied the Kolmogorov–Smirnov test. The significance of differences between groups was computed by t-test or Mann–Whitney test. Airway responsiveness for groups were compared by two-way repeated measures ANOVA, with Bonferroni post-hoc corrections. The Pearson correlation coefficient was applied to estimate the correlation between animals’ behavioral test performances and LFP analysis. P-values < 0.05 were considered statistically significant. The effect size was computed by Cohen’s d.
