# Author Correction: ACC-BLA functional connectivity disruption in allergic inflammation is associated with anxiety

**DOI:** 10.1038/s41598-022-07809-w

**Published:** 2022-03-02

**Authors:** Leila Gholami-Mahtaj, Morteza Mooziri, Kolsoum Dehdar, Maryam Abdolsamadi, Morteza Salimi, Mohammad Reza Raoufy

**Affiliations:** 1 Department of Physiology, Faculty of Medical Sciences, Tarbiat Modares University, Tehran, Iran; 2 School of Medicine, Zahedan University of Medical Sciences, Zahedan, Iran; 3 Department of Mathematics, Faculty of Science, Islamic Azad University-North Tehran Branch, Tehran, Iran; 4 Institute for Brain Sciences and Cognition, Faculty of Medical Sciences, Tarbiat Modares University, Tehran, Iran

Correction to: *Scientific Reports* 10.1038/s41598-022-06748-w, published online 17 February 2022

In the original version of this Article, Maryam Abdolsamadi was incorrectly affiliated with ‘Department of Physiology, Faculty of Medical Sciences, Tarbiat Modares University, Tehran, Iran’. The correct affiliation is listed below.

Department of Mathematics, Faculty of Science, Islamic Azad University-North Tehran Branch, Tehran, Iran.

As a result of the changes, the Affiliations have been renumbered.

The original Article has been corrected.

